# Pericarditis, with Broncho-Pneumonia

**Published:** 1875-02-01

**Authors:** N. S. Davis

**Affiliations:** Prof. Clin. Med.


### Clinical Reports.

### PERICARDITIS WITH BRONCHO-PNEUMONIA.

A Clinical Lecture in the Medical Wards of the Mercy Hospital, by N. S. Davis, A.M., M.D., Prof. Clin. Med.

GENTLEMEN: The patient before you is a native of Ireland; aged about twenty-two years; a laborer; naturally strong and athletic. He was admitted to the hospital six days since, and was reported to have been sick nearly two weeks previously. He had been suffering from severe cough; pain in the chest, aggravated by motion; shortness of breath, and fever.

At the time of his admission, his face was deeply suffused with purplish redness; the cutaneous surface generally, and especially over the extremities, congested and cool; expression of countenance dejected and anxious; respiration short, hurried, and difficult; expectoration scanty, tenacious, and slightly bloody; cough frequent, and suffocative; pulse small, soft, and 120 per minute; tongue covered with a white coat; bowels inactive, and urine scanty. He complained of severe pain and soreness in both sides of his chest, but more severe in the cardiac region than elsewhere.

Percussion revealed entire dullness over the lower and posterior part of the right side of the chest, extending from the axilla to the diaphragm, and from the border of the pectoral muscle to the spine. The area of cardiac dullness was greater than natural, but over the rest of the left side of the chest, the resonance was nearly natural.

Auscultation, detected an unusually loud pericardial friction over the whole cardiac space, with increased impulse, but no unnatural valvular murmurs. A mixture of dry and coarse mucous rhonchi were heard over all the anterior part of the chest, except the cardiac space; but the lateral and posterior part of the right side gave only a sharp, sub-crepitant rale, with apparently very limited inflation. After the members of the class had individually noted the present physical signs, their attention was directed to a careful comparison of these with the general symptoms and history of the case, for the purpose of arriving at a full diagnosis.

The sharp pains, the frequent small pulse with loud pericardial friction, and full impulse, clearly indicated acute-pericarditis, with plastic exudation. The mixture of dry, wheezing, and coarse mucous sounds, with but little alteration of resonance over the anterior and left side of the chest, indicated general bronchitis, while the dullness on percussion, the limited expansion, and the fixed, subcrepitant rale, in the lateral and posterior part of the right side, indicate intense pneumonic engorgement or solidification of the corresponding part of that lung. The length of time the patient had been sick, compared with the present phenomena, rendered it certain that the inflammatory action in the pericardium, bronchia and right lung, was in the second stage of its progress.

The prognosis in this case is decidedly unfavorable. First, the pericardial inflammation may result in so much effusion, both plastic and serous, as to fatally embarrass the action of the heart. Second, the exudation into the right lung may be so copious in consequence of the excited condition of the heart, and the imperfect decarbonization of the blood, as to induce gangrene of some portion of lung tissue coupled with diffuse suppuration, and copious puruloid and fœtid expectoration. Third, the patient may escape both the foregoing perils, and yet become suffocated from œdematous infiltration into the portions of the lungs not involved in pneumonia. When the patient was first admitted to the hospital, the very strongly marked friction sound over the whole cardiac space, coupled with the extreme dyspnœa, caused us to cover his chest with emollient poultices, and give him a powder of calomel, two grains, and bicarbonate of soda and Dover's powder, each five grains, every four hours, and a teaspoonful of the following prescription, between:

℞.—Hydrochlorate of Ammon., ʒ iii.
Sulph. Morph., grs. iii.
Tart. Ant. et. Pot., grs. ii.
Tinct. Digitalis, ℥ i.
Syrup Liquorice, ℥ iii.
Mix.

Under this treatment, the pericardial friction has decidedly diminished, and both pulse and respiration have somewhat diminished in frequency. The danger from the pericardial inflammation is therefore lessening, and is not so much to be dreaded in the future as either of the other important pathological conditions named.

We will now place a blister over the right side of the chest, continue the prescription containing the hydrochlorate of ammonia, one teaspoonful every four hours, and alternate with it, sulphate of quinia, in doses of two or three grains, and feed him regularly with bland nourishment.

The quinia is given for the purpose of increasing the tone of the pulmonary capillaries, and thereby lessening the danger from pulmonary œdema, as well as to exert a general tonic influence. After one week had elapsed, the attention of the class was again called to the same patient.

He had continued the treatment just named during the first half of the week, when the expectoration becoming more copious, thin, purulent, and offensive, while the general bronchial rhonchi and pericardial friction had continued to decrease, two grains of pulv. g. camphor, and one-eighth of a grain of morphia was added to each dose of the quinia, and the hydrochlorate of ammonia mixture was exchanged for the following:

℞.—Liquor Ammon. Acetatis, ℥ ii.

Camph. Tinct. Opii, ℥ ii.

Carb. Ammon., ʒ iss.

Mix. Take one teaspoonful every four hours, in water.

This treatment, with as much nourishment as the patient could be induced to take, has been continued to the present time. The attention of the class was called to the fact, that the pericardial friction had now entirely disappeared, and the cardiac sounds were natural. The bronchial rhonchi had also disappeared from the left side of the chest, and the resonance was nearly natural; while over the whole lateral and posterior part of the right side, there remained entire dullness—no vesicular murmur, but anteriorly a coarse, movable, moist rhonchus. The patient is expectorating large quantities of a thin, muco-purulent matter, emitting a very offensive or putrid odor; his pulse is frequent and weak; his countenance pale and shrunken; and his expression anxious. From the symptoms now presented, it is evident that the bronchial and pericardial inflammations have nearly disappeared, while the pneumonia, involving the middle and lower part of the right lung, has resulted in gangrene of a portion of the inflamed structure, which is now undergoing disintegration, and giving rise to the copious and putrid expectoration. This was one of the dangers pointed out at the previous clinic, and it is now apparent that it will soon terminate the life of the patient. Gangrene of the lungs, as a result of pneumonic inflammation, is certainly of rare occurrence in this locality, this being the first well marked case that has come under our observation in several years.

It is probable, that in the early stage of the case, the exudation from the over-distended capillaries was so copious, as to completely obstruct the circulation through a circumscribed portion of the inflamed tissue, in which case, death of the part must speedily ensue; or at a later period when the circulation was greatly embarrassed by the coincident pericarditis and bronchial obstruction, emboli may have formed in one or more of the arteries of the inflamed lung, plugging them so completely as to cut off the supply of blood to a limited portion of the tissue.

It will be remembered, that the patient had been sick at least one week before his admission to the hospital, and consequently, the first or congestive stage of the pneumonia had already passed, and exudation and hepatization had progressed so far, as to cause well marked dullness on percussion with suppression of the vesicular murmur; and from the extreme dyspnœa, with purple hue of the countenance, cool extremities, soft and quick pulse, it is probable, that if gangrene actually exists, it took place in the manner first mentioned; and it properly suggests the question, whether free bleeding from the arm during the first twenty-four or forty-eight hours after the commencement of the attack, might not have so far lessened the engorgement of the pulmonary vessels, and thereby limited the amount of exudation, as to have prevented the entire obstruction which has followed? From frequent opportunities to observe carefully the effects of prompt bleeding in the first stage of pneumonia, pleuro-pneumonia, and cardiac inflammations, we are as certain that such bleeding is capable of, at least, temporarily relieving or lessening the vascular fullness in such cases, as we are that quinia is capable of interrupting the paroxysms of an intermittent fever.

We cannot, therefore, sanction or justify that extreme professional opinion which would require you to exclude entirely the use of the lancet in medical practice. To be beneficial, however, the bleeding must be practiced in the first stage of the inflammation. After the tissue is already filled with exudative material, outside the capillary vessels, the abstraction of blood by venesection can do no good. At present, it is not likely that any treatment will do more than to palliate the cough, and temporarily sustain the strength of the patient. We will continue to encourage him to take nourishment, with moderate doses of opium and stimulants.

*Note.* — During the twenty-four hours succeeding the time of the last clinical examination, the patient became steadily more exhausted; the sputa more offensive, and of a dark brown color, as if mixed with disintegrating tissue; the mind became wandering; he refused all ingesta, and died on the evening of the third day. Although a *post-mortem* examination was much desired, yet it was refused by relatives, who immediately claimed the body for burial.

